# Transspinal stimulation preceding step training reorganizes spinal locomotor circuits in human spinal cord injury: a randomized sham-controlled clinical trial

**DOI:** 10.3389/fncir.2026.1876368

**Published:** 2026-07-16

**Authors:** Timothy S. Pulverenti, Abdullah M. Sayed Ahmad, Noam Y. Harel, Maria Knikou

**Affiliations:** 1 Klab4Recovery SCI Research Lab, The City University of New York, New York, NY, United States; 2 Department of Physical Therapy, College of Staten Island, The City University of New York, New York, NY, United States; 3 James J. Peters Veterans Affairs Medical Center, Bronx, NY, United States; 4 Icahn School of Medicine at Mount Sinai, New York, NY, United States; 5 PhD Program in Biology and Collaborative Neuroscience Program, Graduate Center of The City University of New York and College of Staten Island, New York, NY, United States

**Keywords:** locomotor training, multimodal interventions, spinal circuits, spinal cord injury, transspinal stimulation

## Abstract

**Introduction:**

Activity-based rehabilitation, such as locomotor training, is commonly used to induce positive systemic physiological adaptations and restore locomotion in individuals with chronic spinal cord injury (SCI). Restoration of locomotion with activity-based rehabilitation alone is not optimal and may require augmentation with neuromodulation strategies.

**Materials and methods:**

We characterized the excitability of spinal locomotor circuits during stepping in humans with SCI who underwent transspinal stimulation before locomotor training, within the same session. A total of 14 participants with chronic SCI received an average of 40 sessions of 30 Hz transspinal stimulation delivered for 30 min during standing (active or sham) or in the supine (active) position, followed by 30 min of robotic-assisted step training. Before and after the completion of all training sessions, we assessed the soleus H-reflex phase-dependent amplitude modulation and reciprocal Ia and presynaptic inhibition in response to a conditioning stimulus delivered to the common peroneal nerve.

**Results:**

Transspinal stimulation administered before locomotor training promoted soleus H-reflex depression during the swing phase in the active standing group (*n* = 5; *F*_1, 309_ = 4.52, *p* = 0.034), stabilized soleus H-reflex excitability during the stance phase in the active supine group (*n* = 5; *F*_1, 327_ = 18.44, *p* < 0.001), and promoted reduced ankle co-contraction during assisted stepping in all groups. Reciprocal inhibition between ankle flexors and extensors after treatment was reduced at mid-stance and during the swing-to-stance transition phase in the active standing group (*n* = 4; *F*_1, 81_ = 7.4, *p* = 0.008), while it was markedly potentiated through the swing phase in the sham standing group (*n* = 3; *F*_1, 108_ = 22.46, *p* < 0.001). Finally, generalized soleus H-reflex depression after the intervention in the active (*n* = 4; *F*_1, 150_ = 42.32, *p* < 0.001) and sham (*n* = 3; *F*_1, 64_ = 13.19, *p* < 0.001) standing groups supported potentiation for presynaptic inhibition.

**Conclusion:**

This study demonstrated that transspinal stimulation preceding locomotor training was associated with modulation of the spinal reflex pathways. These neurophysiological adaptations suggest increased responsiveness of spinal circuits, although further studies are needed to determine whether these changes translate into functional improvements in people with chronic SCI.

## Introduction

The debilitating sequelae of spinal cord injury (SCI) greatly reduce mobility, independence, and quality of life; increase morbidity; and impose large financial burdens on healthcare (Piatt et al., 2016; Chopra and Kumar, 2025; Goulet et al., 2019). Accordingly, the recovery of essential physiological functions that improve ambulation is a priority for the SCI community (Anderson, 2004; Simpson et al., 2012). Considering the lifestyle consequences of SCI and that approximately 300,000 individuals live with SCI in the United States, with an estimated 18,000 new cases annually (National Spinal Cord Injury Statistical Cente, 2024), there is a need to develop rehabilitation strategies and treatments that can alleviate these debilitating deficits while also being widely scalable and cost-effective.

Activity-based rehabilitation, such as locomotor training, is commonly used to induce positive systemic physiological adaptations and restore function in individuals with SCI (Behrman and Harkema, 2007; Harkema et al., 2012; Hubli and Dietz, 2013). Interestingly, among individuals with SCI, locomotor training improves not only walking and standing abilities but also bowel, bladder, and cardiovascular functions (Harkema et al., 2008, 2012; Knikou, 2013; Smith et al., 2015; Hubscher et al., 2018). Unfortunately, recovery with activity-based rehabilitation is not optimal and may require additional neuromodulation strategies, such as electrical stimulation (Behrman et al., 2017; Courtine and Sofroniew, 2019). Notably, painless transspinal (also known as transcutaneous) stimulation can augment the benefits of activity-based rehabilitation (Nardone et al., 2015; Megía García et al., 2020; Pulverenti et al., 2022; Sayed Ahmad et al., 2025).

Transspinal stimulation targeted for locomotor rehabilitation likely affects pre-programmed spinal neural networks [central pattern generators (CPGs)], which coordinate muscle activity during stepping and are modulated by sensory afferent feedback (Côté et al., 2018; Grillner and El Manira, 2020; Grillner and Kozlov, 2021). Extrinsic tonic electrical stimulation over the thoracolumbar enlargement depolarizes a broad range of afferents that produce the transsynaptic activation of leg spinal motor circuits engaged in locomotion in healthy humans or in individuals with SCI (Courtine et al., 2007; Dy et al., 2010). However, because transspinal stimulation and peripheral sensory inputs share common pathways (Knikou and Murray, 2018), transspinal stimulation may interfere with the integral sensory feedback needed to drive motor recovery (Formento et al., 2018). When considered alongside the acute effects of transspinal stimulation, which increase spinal excitability and improve spinal inhibitory circuits (Benavides et al., 2020; Hofstoetter et al., 2020), utilizing transspinal stimulation as a primer to step training may be a beneficial training strategy.

Consequently, we propose that transspinal stimulation, delivered as a “primer” before step training, produces neurophysiological changes consistent with improved excitability of spinal neuronal circuits in individuals with chronic SCI. The objectives of this study were to assess changes in spinal neuronal networks engaged in locomotion when transspinal stimulation over the thoracolumbar enlargement was administered before locomotor training within the same session in individuals with SCI. Active transspinal stimulation was administered during body weight-supported (BWS) standing or resting supine, while sham stimulation was administered during BWS standing. Before and after 40 sessions, we evaluated changes in the phase-dependent amplitude modulation of the soleus H-reflex, reciprocal Ia, and presynaptic inhibition of the soleus H-reflex during stepping. The mechanistic rationale for implementing standing and supine positions was largely based on the state-dependent modulation of spinal circuitry by peripheral sensory afferent feedback. Specifically, plantar cutaneous mechanoreceptors and weight-bearing load receptors (Ib afferents from Golgi tendon organs) are activated during standing (Pratt, 1995; Capaday et al., 1995; Lavoie et al., 1997). This specific afferent input has been shown to gate spinal interneuronal pathways, facilitating extensor motor pool activity and modulating the CPG circuitry in a manner that the non-weight-bearing (supine) position cannot (Conway et al., 1987). Furthermore, a tonic stretch of hip and ankle extensors during upright standing provides continuous Ia and II afferent feedback to the lumbar segments. Delivering transspinal stimulation concurrently with this posture-specific sensory input may lower the activation threshold of paralyzed or paretic motor networks, leading to a synergistic rather than an additive effect (Sayenko et al., 2019). Finally, upright posture engages vestibular and tonic postural descending pathways, which, even if partially disrupted post-SCI, alter the excitability state of the spinal cord compared to the supine position (Horak and Nashner, 1986; Deliagina et al., 2012; Lemay et al., 2015; Milosevic et al., 2017a,b). We hypothesized that the function of spinal locomotor circuits would improve more in the active standing group than in the other groups.

## Materials and methods

### Participants

People with American Spinal Injury Association Impairment Scale (AIS) grade B, C, or D lesions at or rostral to the Thoracic 10 neurological spinal level participated in this clinical trial. The detailed inclusion and exclusion criteria are available in the published clinical trial protocol (Skiadopoulos et al., 2023). Each participant provided a signed informed consent before study enrollment and participation. The clinical trial was approved by the Institutional Review Board (IRB) of the City University of New York (CUNY Biomedical IRB 2019-0806) and the James J. Peters Veterans Affairs Medical Center (JJPVAMC IRBNet 01919). All study procedures were conducted in compliance with the Declaration of Helsinki. A total of 14 participants completed the trial protocol. Eight participants completed the training sessions at the Klab4Recovery SCI Research Lab at CUNY and six at JJPVAMC. All neurophysiological assessments were completed at Klab4Recovery. The trial is registered on ClinicalTrials.gov (ID NCT04807764). Subjects’ demographics are shown in Table 1.

**Table 1:** Demographics and injury characteristics of participants with chronic spinal cord injury (SCI).

Subject ID	Gender	Age	Vertebra level of injury	AIS^a^ scale	Time post injury (year)	Cause of injury	Neurotropic medications	Number of sessions attended
Active standing								
NIH007	F	43	C1	D	2	MVA	Baclofen 10 mg 5 times/day, cyclobenzaprine 15 mg/day	41
							Dentrolene 50 mg 3 times/day	
							Duloxetin 20 mg 2 times/day	
NIH008	F	25	T4	D	10	Scoliosis repair surgery	None	41
NIH009	M	67	C3	D	3	Fall in the bathroom	Baclofen 35 mg/day, gabapentin 300 mg 3 times/day, pregabalin 150 mg 3 times/day, diazepam 0.5 mg 3–4 times/day,	41
NIH011	M	43	T5	D	2	Fall from the roof	Baclofen 5 mg 5 times/day, dalfampridine 10 mg 2 times/day	43
Sham standing								
NIH001	M	60	C2	B	11	Swimming	Baclofen 20 mg 1 time/day	40
NIH002	F	61	C4	D	10	Swimming	Baclofen 20 mg 2 times/day, cyclobenzaprine 5 mg 3 times/day, oxybutynin 10 mg/day, amitriptyline 10 mg/day	40
NIH003	M	53	T3	D	12	Spinal arachnoid cyst	Baclofen 20 mg 4 times/day; testosterone 40.5 mg 2 times/week	40
NIH010	M	23	T10	B	3	Ski accident	Baclofen 10 mg 3 times/day, gabapentin 300 mg 2 times/day	42
NIH014	M	29	T8	D	15	AVM^d^ rupture	Methenamine hippurate 1 g 2 times/day	41
Active supine								
NIH004	F	34	C3	D	17	MVA^c^	Bupropion 300 mg 1 time/day	40
NIH005	M	47	T8	D	22	Gunshot	Oxycodone 10 mg, gabapentin 300 mg	34
NIH006	F	28	T2	B	8	Gunshot	Vibegron 75 mg 1 time/day	36
NIH012	M	57	T3	D	1	Transverse myelitis	Baclofen 25 mg 3 times/day, Tizanidine 4 mg 3 times/day, Oxybutinin 10 mg 1 time/day	41
NIH013	M	25	C4	D	0.5	Spinal stroke	Myrbetriq 15 mg 1 time/day, Atorvastatin 20 mg 1 time/day	36
							Cialis 2.5–5 mg as needed	

a American Spinal Injury Association Impairment Scale.

^b^All groups received 30 min of locomotor training with the Lokomat 6 Pro after 30 min of active or sham transspinal stimulation during supine or standing.

c Motor vehicle accident.

d Arteriovenous malformations.

### Intervention: transspinal stimulation before locomotor training within the same session

Transspinal stimulation was delivered for 30 min before locomotor training for all participants. Participants were randomized into three groups. Active or sham transspinal stimulation was delivered in the BWS standing position, and active transspinal stimulation while resting supine. Detailed information on the transspinal stimulation intensities for each participant across sessions is published in Sayed Ahmad et al. (2025). Participants did not receive concurrent rehabilitation other than weight-bearing standing in two of the groups. We disclosed to all participants before enrolment that they may be randomized to active or sham stimulation. During the study, participants were not provided information on which group they were assigned to. Participants were disclosed of their assigned group after the study had ended. However, their perceptiveness regarding the type of stimulation (active vs. sham) at the end of the study was not assessed.

A constant-current stimulator (DS8R, Digitimer Ltd., UK) was used to deliver 1.0-ms bursts composed of a charge-balanced, symmetric, biphasic rectangular 10-kHz carrier frequency at 30 Hz for 30 min per session. To account for daily variations in electrode-to-skin impedance and spinal cord excitability, the paresthesia threshold was reassessed at the beginning of every session. With the participant in the assigned standing or supine position, stimulation intensity was progressively increased until the participant first reported a clear, bilateral tingling sensation in the anterior/posterior hips and thighs. The stimulation intensity for each session was then set to 1.2 times the daily threshold.

Transspinal stimulation was delivered at 30 Hz because, at this frequency, step-like or synchronous rhythmic movements have been reported in both non-injured and SCI subjects (Gerasimenko Y. et al., 2015; Gerasimenko Y. P. et al., 2015; Danner et al., 2015). Moreover, the transspinal stimulation intensity was largely based on each participant’s comfort level. Nevertheless, transspinal stimulation at intensities both above and below the motor threshold intensities produces notable neurophysiological changes after multiple sessions in both animals and humans (Murray and Knikou, 2019; Malloy et al., 2025).

A single reusable self-adhesive cathode electrode (10.2 × 5.1 cm^2^, Uni-Patch, MA, USA) was placed at the midline, overlying the vertebrae equally between the left and right paravertebral sides, covering the Thoracic 10 to Lumbar 1–2 vertebral levels. The anode consisted of a pair of interconnected electrodes (the same type as the cathode) placed on each iliac crest. Our configuration of the cathode and anode electrodes requires less stimulation intensity to depolarize alpha motoneurons while maximally engaging spinal inhibitory mechanisms (Skiadopoulos et al., 2022). The Thoracic 10 spinous process was identified via palpation and based on anatomical landmarks (the end of the rib cage and the Thoracic 1 vertebra). Consistent cathodal electrode position across intervention sessions was ensured by carefully recording the electrode’s position relative to anatomical landmarks. For participants who received transspinal stimulation while lying supine, the hips and knees were placed in slight flexion and stabilized with holsters and towels, as needed, to avoid bilateral external limb rotations.

Active transspinal stimulation was delivered during standing under BWS on the Lokomat 6 Pro® (Hocoma, Switzerland). BWS during standing was adjusted to minimize knee buckling and reached an average of 19% in the sham standing and 45% in the active standing. Sham transspinal stimulation during standing consisted of current delivery at above the paresthesia threshold for 1 min, ramped slowly to 0 mA intensities that remained for 28 min, followed by ramping back to the above paresthesia threshold for the final minute, similar to sham methods used in previous studies (Benavides et al., 2020; Hofstoetter et al., 2020; Spieker et al., 2025; Bye et al., 2026).

Locomotor training for all participants was performed 5 days per week for 36–43 sessions. The duration of locomotor training per session was 30 min of stepping in the robotic gait orthosis Lokomat 6 Pr ® (Hocoma, Switzerland). The BWS, leg guidance force (LGF), treadmill speed, and ankle strap tension were adjusted to eliminate toe and foot dragging and knee buckling during assisted stepping. As participants progressed through the intervention, these variables were adjusted, where appropriate, using a clinical algorithm we have previously utilized for locomotor training trials in humans with SCI (Knikou, 2013; Smith et al., 2015). Detailed information on the BWS, LGF, and treadmill speed data for each participant across the training sessions is published in Sayed Ahmad et al. (2025).

### Experimental procedures for neurophysiological measurements

The investigators performing neurophysiological assessments and data analyses were blinded to treatment allocation and the study group to which each participant was enrolled.

#### Surface electromyography

Surface electromyographic (EMG) electrodes were used to record control and conditioned soleus H-reflexes and soleus (SOL) and tibialis anterior (TA) locomotor EMG activity. The skin was dry-shaved, abraded, and cleaned with alcohol before surface EMG electrode placement. Differential bipolar electrodes (2 cm interelectrode difference; Motion Lab System EMG preamplifier) were placed over each muscle belly and secured with Tegaderm transparent film (3 M Healthcare, St. Paul, MN, USA). All EMG data were recorded at a 2,000 Hz sampling rate via an EMG unit (MA300 DTU, Concord Motion Labs, Knoxville, TN, USA) and acquired using a 16-bit data acquisition card (NI-PCI-6225, National Instruments, Austin, TX, USA) running a customized LabVIEW software interface during standing and stepping data collection.

#### Peripheral nerve stimulation

##### Posterior tibial nerve

While each subject was seated, a stainless-steel plate anode electrode of 4 cm in diameter was placed and secured proximally to the patella. Rectangular single-pulse stimuli of 1-ms duration at 0.2 Hz were delivered by a constant current stimulator to the tibial nerve at the popliteal fossa. The optimal stimulation site was established using a hand-held monopolar stainless-steel head electrode as a probe (Knikou, 2008). An optimal stimulation site corresponded to the site at which the M-wave had a shape similar to that of the H-reflex at low and high stimulation intensities. At the lowest stimulus intensity, an H-reflex could be evoked without an M-wave. When the optimal site was identified, the monopolar cathode electrode was replaced with a pregelled disposable electrode (SureTrace, Conmed, Utica, NY, USA), which was maintained under constant pressure throughout the experiment using an athletic wrap.

##### Common peroneal nerve (CPN)

The CPN was stimulated by a bipolar stainless-steel electrode placed distal to the head of the fibula via monophasic single-pulse stimuli of a duration of 1 ms at 0.2 Hz. The optimal stimulation site corresponded to the one where, at increased levels of intensities, the peroneus longus muscle was silent, the TA motor threshold (MT) was consistently lower than that of the peroneus longus muscle, and at increased stimulation intensities, selective ankle dorsiflexion without ankle eversion was induced (Knikou, 2005, 2008). The CPN was stimulated with a constant current stimulator (DS7A, Digitimer, UK). The stimulus to the CPN was delivered at a threshold of 0.9–1.2 TA M-waves across subjects.

Reciprocal inhibition, assessed via CPN conditioning stimulation of the soleus H-reflex, is delivered at 0.9–1.0 times the TA M-wave threshold. In contrast, the strength of reciprocal inhibition depends on the strength of the conditioning stimulus (Crone et al., 1985, 1987, 1994). In this study, for each patient, before recording, we established at baseline whether (1) reciprocal inhibition was replaced by reciprocal facilitation and (2) reciprocal facilitation reversed to reciprocal inhibition when the conditioning stimulus strength was increased at a constant TA M-wave amplitude (data not shown). When this was identified, we selected the multiples of the TA M-wave threshold at which the CPN was stimulated. The TA M-wave was monitored during the experiment to ensure consistency of the conditioning stimulation. For each subject, the conditioning stimulus after training was delivered at similar multiples of the TA M-wave threshold utilized before training.

#### Neurophysiological recordings during stepping

Control and conditioned soleus H-reflexes during BWS-assisted stepping were recorded before (baseline) and 1–2 days after the last training session for each participant, using the individualized Lokomat settings (e.g., BWS, leg guidance, and treadmill speed) used at baseline.

After the optimal stimulation sites for mixed peripheral nerves were established with the subjects seated, each subject was transferred to the treadmill and was fitted with an upper body harness connected to the Lokomat’s BWS support system. The thigh and shank segments of the robotic device were adjusted based on each subject’s leg length and diameter, and both feet were secured into the foot lifters. With the subject standing at a BWS that prevented knee buckling, a full soleus H-reflex and M-wave recruitment curve was first obtained using approximately 80 stimuli delivered at 0.2 Hz (Knikou et al., 2009a,b). The recruitment curve information regarding stimulation intensities and amplitudes of the M-wave and H-reflex was saved and used to determine the most “optimal” stimulation intensity during stepping.

During BWS-assisted stepping, we used a custom-built constant-current stimulator in which the stimulus intensity is computer-controlled and adjusted based on a custom machine-learning algorithm built in LabVIEW. Once each participant began to step with BWS, the stimulation intensities and amplitudes from the recruitment curves were retrieved, and the optimal intensity was identified. This optimal intensity was set to evoke an H-reflex on the ascending limb of the recruitment curve because of the susceptibility of the H-reflex to facilitation and inhibition based on its size (Crone et al., 1990), while an M-wave ranging from 2 to 15% of the maximal M-wave was maintained to ensure constant stimulation intensity targeting the posterior tibial nerve during movement (Knikou et al., 2009a; Knikou and Mummidisetty, 2011). In real time, the algorithm calculated the amplitudes of the M-wave and H-reflex as percentages of the maximal M-wave evoked 80 ms after each H-reflex stimulus. When the test response was rejected based on the M-wave amplitude, the algorithm adjusted the stimulation intensity to evoke H-reflexes whose associated M-waves were within the acceptable range. This was carried out randomly across the different bins of the step cycle until a full step-cycle modulation pattern was recorded. Data acquisition continued until at least five accepted H-reflexes were obtained at each bin of the step cycle. This procedure was adopted for recordings of both control and conditioned H-reflexes.

The soleus H-reflex depression by CPN stimulation at short conditioning-test (C-T) intervals (mainly 1–3 ms) reflects the amount of disynaptic reciprocal Ia inhibition exerted postsynaptically from TA inhibitory interneurons activated by TA afferents onto soleus motoneurons with a 0.9–1.0 ms central delay (Mizuno et al., 1971; Ashby and Labelle, 1977; Ashby and Zilm, 1978; Day et al., 1984; Crone et al., 1987; Knikou, 2008). Presynaptic inhibition of Ia afferents evoked by the conditioning stimulation of the antagonistic muscle afferents starts approximately at 10 ms C-T interval and lasts up to 1,000 ms (Hultborn et al., 1987; Capaday et al., 1995; Faist et al., 1996; Knikou et al., 2006; Jessop et al., 2013). During assisted stepping, the shortest (0–3 ms) and medium-latency (20–100 ms) C-T intervals at which absent reciprocal facilitation or reflex inhibition were observed with subjects seated were used (Sayed Ahmad et al., 2025). The TA motor threshold was re-established during BWS standing, while during stepping, the CPN was stimulated at 0.9–1.2 multiples of the TA motor threshold.

In all subjects, stimulation of the mixed peripheral nerves was triggered based on the signal from the ipsilateral foot switch (RX111 and Biopac IPS100C; Biopac Systems, Inc., Goleta, CA, USA). Stimuli were delivered randomly across the different phases of the step cycle. The step cycle was divided into 16 equal bins, which provides high resolution for neurophysiological measures during human stepping (Simonsen et al., 1995; Knikou, 2013; Knikou et al., 2009a). Bin 1 corresponds to heel contact. Early- mid-, and late-stance phases correspond approximately to bins 1–3, 4–6, and 7–9, respectively. Bins 9, 10, and 16 correspond approximately to stance-to-swing transition, swing phase initiation, and swing-to-stance transition, respectively.

### Data analysis

For all recordings during BWS-assisted stepping, the stimulation pulses and the right- and left-foot switches were first marked. The M-waves and H-reflexes, the control and conditioned responses, were identified in each bin of the step cycle using custom LabVIEW scripts. The soleus M-waves and H-reflexes were measured as peak-to-peak amplitudes at each bin of the step cycle and expressed as a percentage of the maximal M-wave evoked 80 ms after the associated test pulse. Accepted soleus H-reflexes, based on the amplitude of the M-wave as a percentage of the maximal M-wave, were grouped per subject group, time, and bin of the step cycle.

The soleus background EMG activity at each bin of the step was estimated as the integrated area of the soleus linear EMG envelope (band-pass filtered 40–500 Hz, low-pass filter of the EMG envelope at 20 Hz, second order polynomial) beginning 100 ms before the posterior tibial nerve stimulation pulse for 50 ms. It was normalized to the maximal soleus EMG activity. For each subject, the mean amplitude of the normalized soleus H-reflex from each bin of the step was plotted on the *y*-axis (dependent variable) against the normalized SOL background EMG activity (independent variable) on the *x*-axis, and a linear regression line was fitted. This was performed before and after each intervention, as well as for pooling data from each subject group. The slope and *y*-intercept from the linear regression were grouped by time and subject group. Statistically significant differences between linear regression slopes and *y*-intercepts were evaluated with a paired *t*-test.

For each subject, the linear EMG envelope (full-wave rectified, band-pass filtered 20–1,000 Hz, low-pass filter of the EMG envelope at 20 Hz, second order polynomial) for the SOL and TA muscles was calculated and then averaged across subjects per study group. To evaluate inter-muscular coordination, locomotor EMG probability distribution plots were constructed for the SOL and TA muscle pairs across the gait cycle (Knikou and Mummidisetty, 2014). Time-normalized and amplitude-normalized EMG envelopes from SOL and TA were plotted against each other point-by-point (2000 points per cycle) from at least 400 steps. A co-activation index was not computed at this stage of data analysis. Concentration along the diagonal axis represents simultaneous, proportional activation of both muscles (antagonist co-activation). Conversely, tight clustering along the individual horizontal TA or vertical SOL axes indicates reciprocal activation strategies.

A two-way-repeated measures analysis of variance (rmANOVA) was performed on the soleus H-reflex recorded for each study group, with factors of time and bins of the step cycle. Three-way rmANOVA was performed separately for reciprocal and presynaptic inhibition with factors of conditioned and unconditioned reflexes, bins, and time of testing. When a statistically significant effect was found, Holm-Sidak pairwise multiple comparisons were applied to the data. In all statistical tests, alpha was set at 0.05. When between-group factors (subject group, time, and bins) for each neurophysiological outcome were included in a mixed linear model, the software produced an error warning when calculating the between-group interaction parameters because the model was overparameterized for the data. As these structural issues compromised the validity of the fixed-effect interaction terms (group, time, and bins), we restricted our primary interpretations to within-group longitudinal profiles where model diagnostics were strictly satisfied. Results are presented as mean values with standard deviation (SD).

## Results

### Changes in soleus H-reflex phase-dependent modulation during assisted stepping

**Figure 1:**
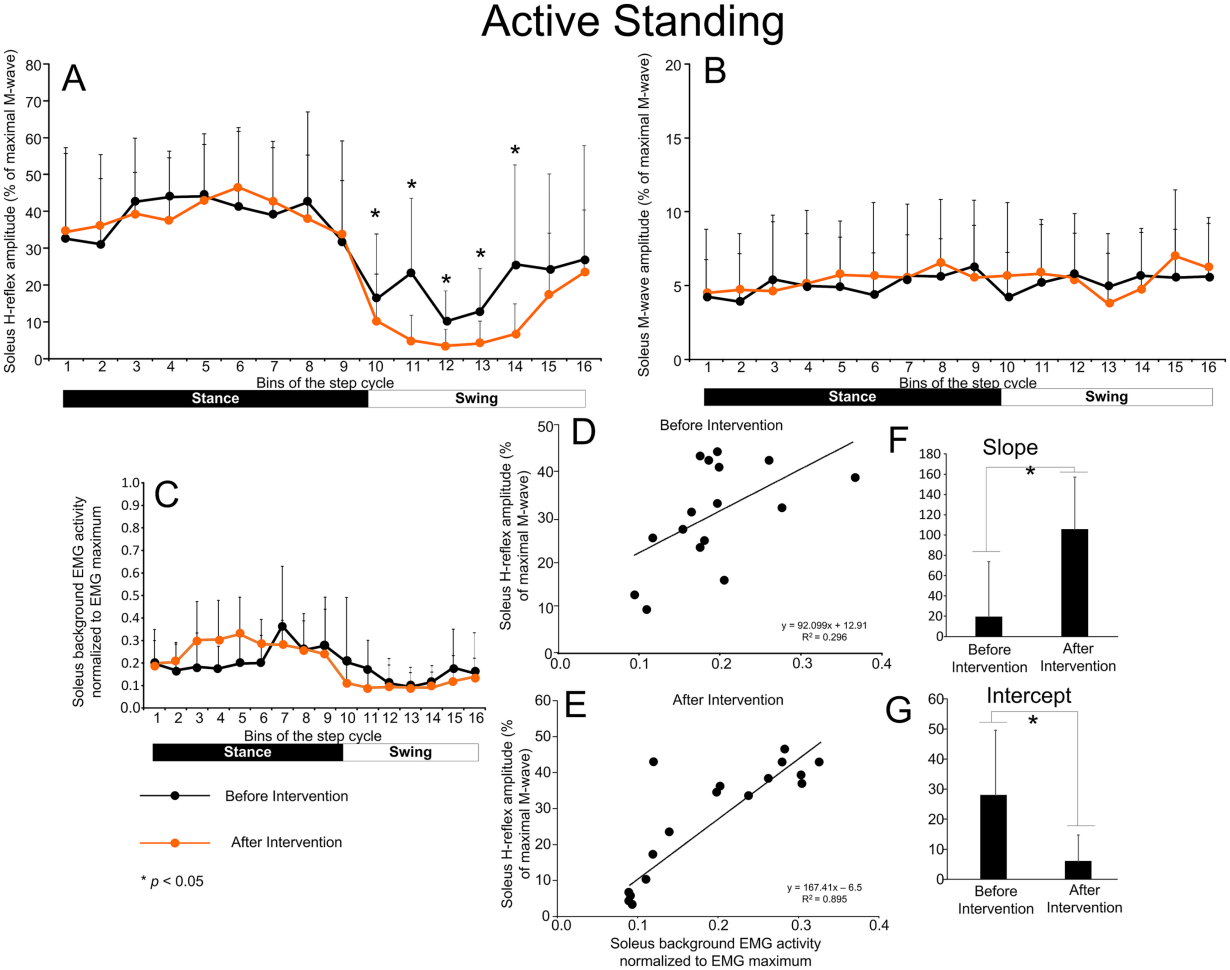
Soleus H-reflex phase-dependent modulation in the active standing group. Mean normalized soleus H-reflex **(A)** and M-wave **(B)** amplitude before (black lines) and after (orange lines) active transspinal stimulation during standing preceding locomotor training from all subjects. SOL normalized EMG background activity **(C)**, along with the normalized soleus H-reflex amplitude plotted against the SOL normalized EMG background activity from all subjects **(D,E)**. The 16 points in graphs **(D)** and **(E)** correspond to the 16 bins of the step cycle. The overall amplitude of the slope **(F)** and intercept **(G)** from all subjects was estimated from the linear relationship between the mean amplitude of the soleus H-reflex and EMG background activity performed for each subject and time. Error bars indicate the SD. ^*^p < 0.05.

Figure 1A shows the average normalized amplitude of the soleus H-reflex recorded during assisted stepping at each bin of the step cycle from all subjects (*n* = 4) before and after *active* transspinal stimulation *while standing* preceding locomotor training. The soleus H-reflex amplitude was significantly different across the step cycle (*F*_15, 309_ = 11.62, *p* < 0.001; $\eta_{p=}^{2}$ 0.36) and between time of testing (*F*_1, 309_ = 4.52, *p* = 0.034; $\eta_{p=}^{2}$ 0.05). Holm-Sidak pairwise multiple comparisons showed a significant decrease in soleus H-reflex amplitudes after the intervention at bins 10–14 (*p* < 0.001). The changes in soleus H-reflex amplitudes occurred with stable M-waves (Figure 1B) recorded across all bins (*F*_15, 309_ = 0.61, *p* = 0.86; $\eta_{p=}^{2}$ 0.029) before and after treatment (*F*_1, 309_ = 0.45, *p* = 0.5; $\eta_{p=}^{2}.0014$ ). The soleus H-reflex amplitude while stepping from all subjects was moderately linearly related to the SOL background EMG activity before treatment (*R*^2^ = 0.29; Figures 1C,D), which changed dramatically after treatment (*R*^2^ = 0.89; Figure 1E). The slope (Figure 1F) and intercept (Figure 1G) between the soleus H-reflex amplitude and soleus background EMG activity significantly increased (*t*_19_ = −4.016, *p* < 0.001). It decreased (*t*_19_ = 3.0, *p* = 0.004), respectively, after treatment, indicating altered soleus H-reflex gain and threshold.

**Figure 2:**
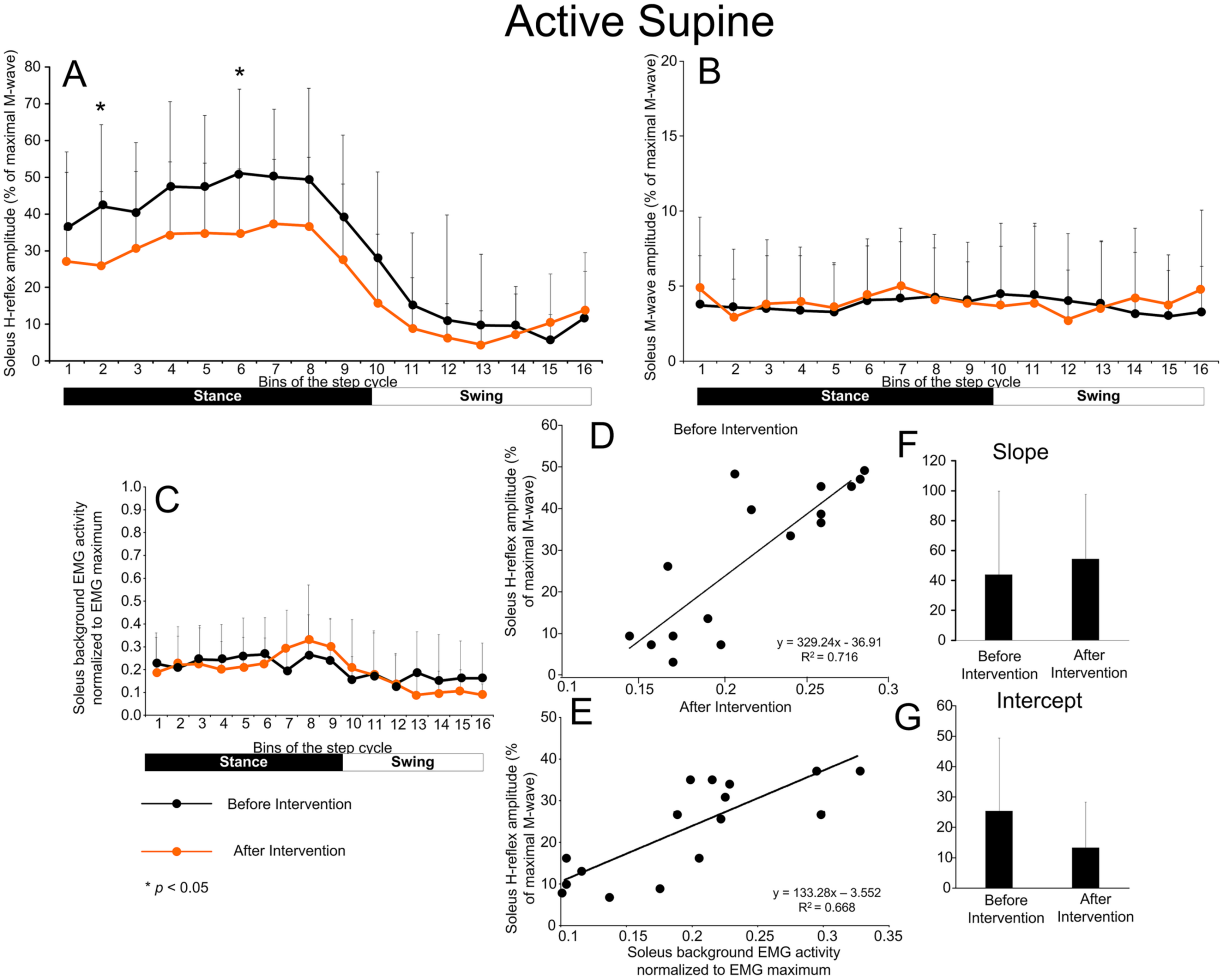
Soleus H-reflex phase-dependent modulation in the active supine group. Mean normalized soleus H-reflexes **(A)** and M-waves **(B)** amplitudes before (black lines) and after (orange lines) active transspinal stimulation during supine preceding locomotor training from all subjects. SOL normalized EMG background activity **(C)**, along with the normalized soleus H-reflex amplitude plotted against the SOL normalized EMG background activity **(D,E)**. The 16 points in graphs **(D)** and **(E)** correspond to the 16 bins of the step cycle. The overall amplitude of the slope **(F)** and intercept **(G)** across all subjects was estimated from the linear relationship between the mean amplitude of the soleus H-reflex and EMG background activity from each subject, and the data were grouped by testing time. Error bars indicate the SD.

Figure 2A shows the average normalized amplitude of the soleus H-reflex recorded during assisted stepping at each bin of the step cycle before and after the intervention from all subjects (*n* = 5) before and after *active* transspinal stimulation *at rest* preceding locomotor training. The soleus H-reflex amplitude was significantly different across the step cycle (*F*_15, 327_ = 13.37, *p* < 0.001; $\eta_{p=}^{2}$ 0.38) and between time of testing (*F*_1, 327_ = 18.44, *p* < 0.001; $\eta_{p=}^{2}$ 0.05). Holm-Sidak pairwise multiple comparisons showed a significant decrease in soleus H-reflex amplitudes after treatment at bins 2 and 6 (*p* < 0.001). The M-wave amplitudes from all subjects (Figure 2B) were similar across all bins (*F*_15, 363_ = 0.29, *p* = 0.99; $\eta_{p=}^{2}$ 0.01) before and after the intervention (*F*_1, 363_ = 0.248, *p* = 0.61; $\eta_{p=}^{2}$ 0.0006). The soleus H-reflex amplitude during stepping was linearly related to the SOL background EMG activity before (*R^2^* = 0.71; Figures 2C,D) and after (*R^2^* = 0.66; Figure 2E) treatment. The slope (Figure 2F; *t*_20_ = −0.203, *p* = 0.42) and intercept (Figure 2G; *t*_20_ = 1.27, *p* = 0.1) between the soleus H-reflex amplitude and soleus background EMG activity did not change after treatment in this group.

**Figure 3:**
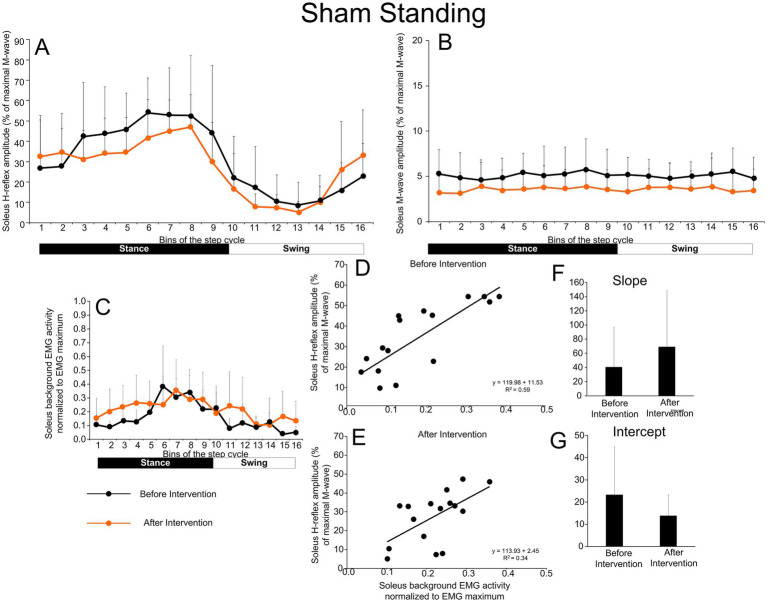
Soleus H-reflex phase-dependent modulation in the sham group. Mean normalized soleus H-reflexes **(A)** and M-waves **(B)** before (black lines) and after (orange lines) *inactive (or sham)* transspinal stimulation *while standing* preceding locomotor training from all subjects. SOL normalized EMG background activity **(C)**, along with the normalized soleus H-reflex amplitude plotted against the SOL normalized EMG background activity **(D,E)**. The 16 points in graphs **(D)** and **(E)** correspond to the 16 bins of the step cycle. The overall amplitude of the slope **(F)** and intercept **(G)** across all subjects was estimated from the linear relationship between the mean amplitude of the soleus H-reflex and EMG background activity from each subject, presented by time of testing. Error bars indicate the SD. ^*^p < 0.05.

Figure 3A shows the average normalized amplitude of the soleus H-reflex recorded during assisted stepping at each bin of the step cycle from all subjects (*n* = 3) before and after *sham* transspinal stimulation *during standing* preceding locomotor training. The soleus H-reflex amplitude was significantly different across the step cycle phases (*F*_15, 206_ = 9.32, *p* < 0.001; $\eta_{p=}^{2}$ 0.4) but not between time of testing (*F*_1, 206_ = 0.07, *p* = 0.79; $\eta_{p=}^{2}$ 0.01). The soleus H-reflex recordings before and after treatment occurred at stable soleus M-wave amplitudes (Figure 3B) across the different bins of the step cycle (*F*_15, 207_ = 0.1, *p* = 1.0; $\eta_{p=}^{2}$ 0.007) at both times of testing (*F*_1, 307_ = 0.7, *p* = 0.45; $\eta_{p=}^{2}$ 0.1). The soleus H-reflexes during BWS-assisted stepping were moderately linearly related to the SOL background EMG activity before intervention (*R*^2^ = 0.59; Figures 3C,D), which remained largely unchanged after the intervention (*R*^2^ = 0.34; Figure 3E). The slope (Figure 3F; *t*_13_ = −0.79, *p* = 0.22) and intercept (Figure 3G; *t*_13_ = 1.11, *p* = 0.14) between the soleus H-reflex amplitude and the soleus background EMG activity remained unaltered after the intervention.

### Changes in reciprocal Ia inhibition during assisted stepping

**Figure 4:**
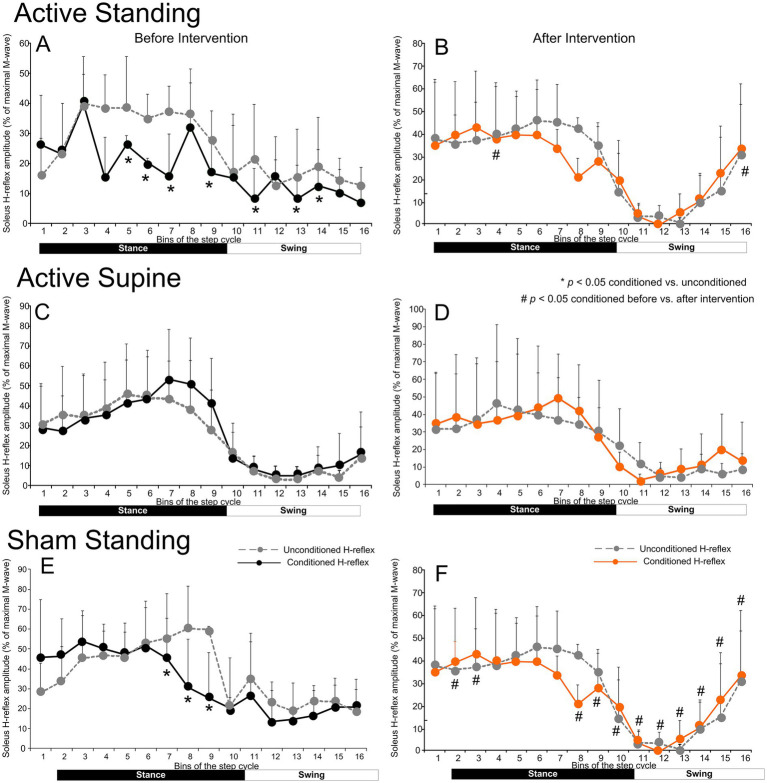
Reciprocal inhibition of soleus Ia afferents before and after each intervention. The mean amplitudes of the conditioned and unconditioned soleus H-reflexes recorded before and after each intervention during stepping are shown as a percentage of the associated maximal M-wave elicited after the unconditioned or test H-reflex at each bin of the step cycle from all subjects. The step cycle was divided into 16 equal bins. Bin 1 corresponds to heel contact. Bins 9, 10, and 16 correspond approximately to stance-to-swing transition, swing phase initiation, and swing-to-stance transition, respectively. Asterisks and hashtags indicate statistically significant differences between conditioned and unconditioned H-reflexes and between conditioned H-reflexes before and after each intervention, respectively. Error bars in all graphs denote the SD.

Figure 4 shows the average amplitude modulation across all subjects tested at each study group of the soleus H-reflex conditioned by CPN stimulation at short C-T intervals, for all 16 bins of the step cycle, reflecting the amount of reciprocal inhibition.

Results for the active standing group (*n* = 4) are shown in Figures 4A,B. The conditioned H-reflex amplitudes were significantly different from the unconditioned reflexes (*F*_1, 155_ = 4.27, *p* = 0.04; $\eta_{p=}^{2}$ 0.026) at bins 5–12, and at bins 14 and 16 (Holm-Sidak; *p* < 0.05), as a function of time (*F*_1, 155_ = 5.51, *p* = 0.02; $\eta_{p=}^{2}$ 0.034), and bins of the step (*F*_15, 155_ = 7.26, *p* < 0.001; $\eta_{p=}^{2}$ 0.41). The conditioned H-reflexes were significantly different across bins (*F*_15, 81_ = 3.48, *p* < 0.001; $\eta_{p=}^{2}$ 0.39) and between time of testing (*F*_1, 81_ = 7.4, *p* = 0.008; $\eta_{p=}^{2}$ 0.083) at bins 4 and 16 (Holm-Sidak; *p* < 0.05), suggesting a phase-dependent amplitude modulation of the conditioned H-reflex and a decrease in reciprocal inhibition at mid-stance and the swing-to-stance transition.

For the active supine group (*n* = 4), the conditioned H-reflexes were significantly different across bins (*F*_15, 139_ = 7.94, *p* < 0.001; $\eta_{p=}^{2}$ 0.46) but not between time of testing (*F*_1, 139_ = 0.01, *p* = 0.89; $\eta_{p=}^{2}$ 0.00013). Further, conditioned H-reflex amplitudes were not significantly different from the unconditioned reflexes (*F*_1, 249_ = 0.56, *p* = 0.45; $\eta_{p=}^{2}$ 0.0022).

Finally, for the sham standing group (*n* = 3), the conditioned H-reflexes were not significantly different from the unconditioned H-reflexes (*F*_1, 108_ = 2.33, *p* = 0.12; $\eta_{p=}^{2}$ 0.021) but were significantly different across bins (*F*_15, 108_ = 7.64, *p* < 0.001; $\eta_{p=}^{2}$ 0.51) and between time of testing (F_1, 108_ = 22.46, *p* < 0.001; $\eta_{p=}^{2}$ 0.17) at bins 2 and 3 and at bins 8–16 (Holm-Sidak; *p* < 0.05; Figure 4F), suggesting an increase in reciprocal inhibition during the swing phase after the intervention. The conditioned H-reflexes after treatment were significantly different across bins (*F*_15, 62_ = 5.18, *p* < 0.001; $\eta_{p=}^{2}$ 0.55) and smaller compared to the conditioned H-reflexes before treatment (*F*_1,62_ = 12.9, *p* < 0.001; $\eta_{p=}^{2}$ 0.17) at bins 2 and 3 (Holm-Sidak; *p* < 0.05).

**Figure 5:**
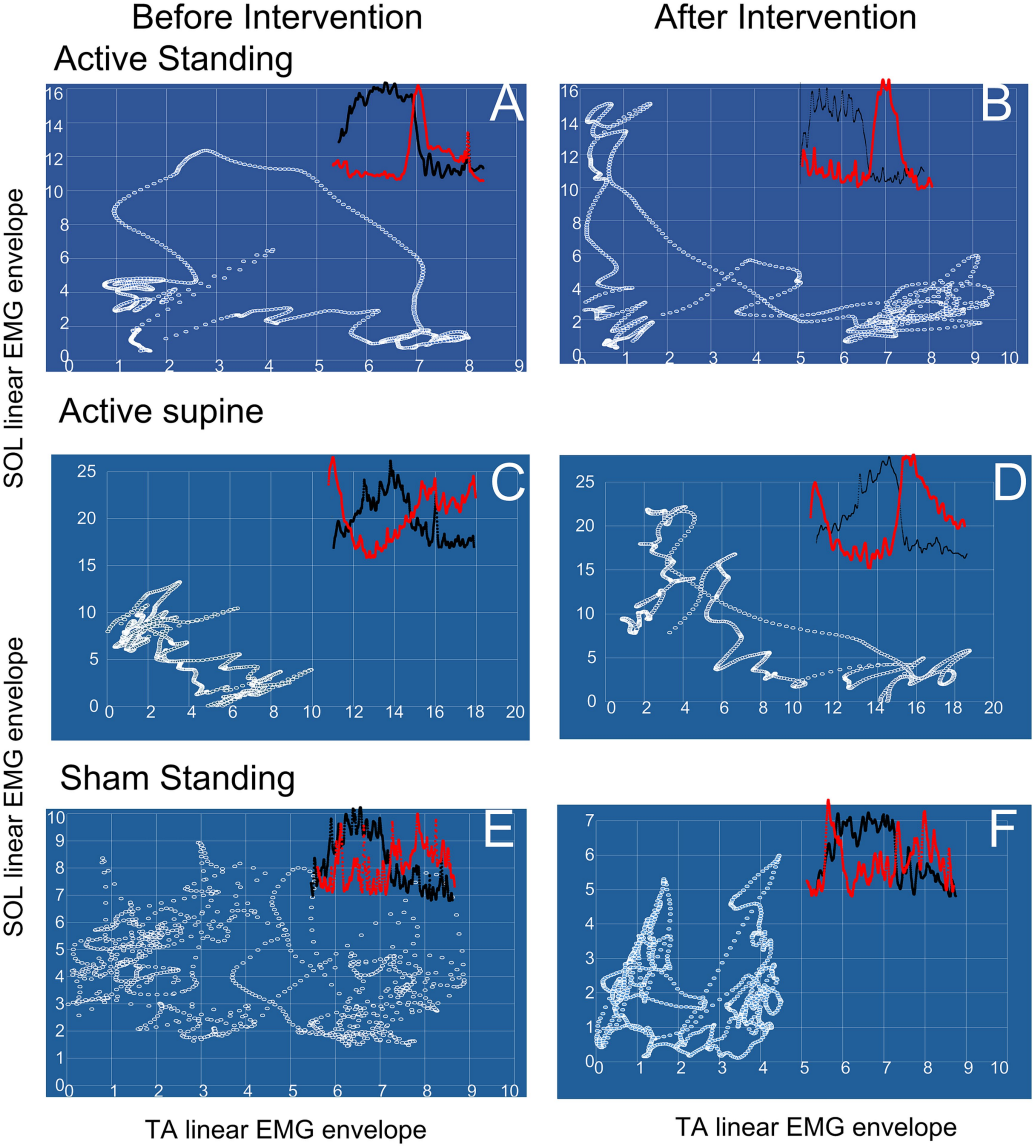
Mean probability distribution plots of the EMG from the SOL and TA muscles before and after each intervention from all subjects for each study group. An L-shaped pattern indicates alternating activity between the two antagonistic ankle muscles. A circular or other shape indicates less alternation and more co-activation. The linear EMG envelopes across the step cycle are also shown for the SOL (black lines) muscle, superimposed on the TA (red lines), before and after each intervention. SOL, soleus; TA, tibialis anterior.

The locomotor probability distribution plots of SOL EMG against TA EMG before and after treatment for each study group are shown in Figure 5. In the active standing group pre-intervention, co-contraction of ankle antagonists was mostly evident (Figure 5A). In contrast, the pattern adopted a more physiological L-shape after the intervention (Figure 5B). This is also reflected in the more reciprocal activation of the muscles (see the insets in Figure 5). Further, improvements were also observed in the active supine group, with more reciprocal activation and discrete activation of the ankle antagonists after the intervention (Figures 5C,D). Finally, in the sham standing group, the physiological “L shape” of SOL/TA EMG probability plots was completely distorted both before and after the intervention (Figures 5E,F).

### Changes in premotoneuronal control during assisted stepping

**Figure 6:**
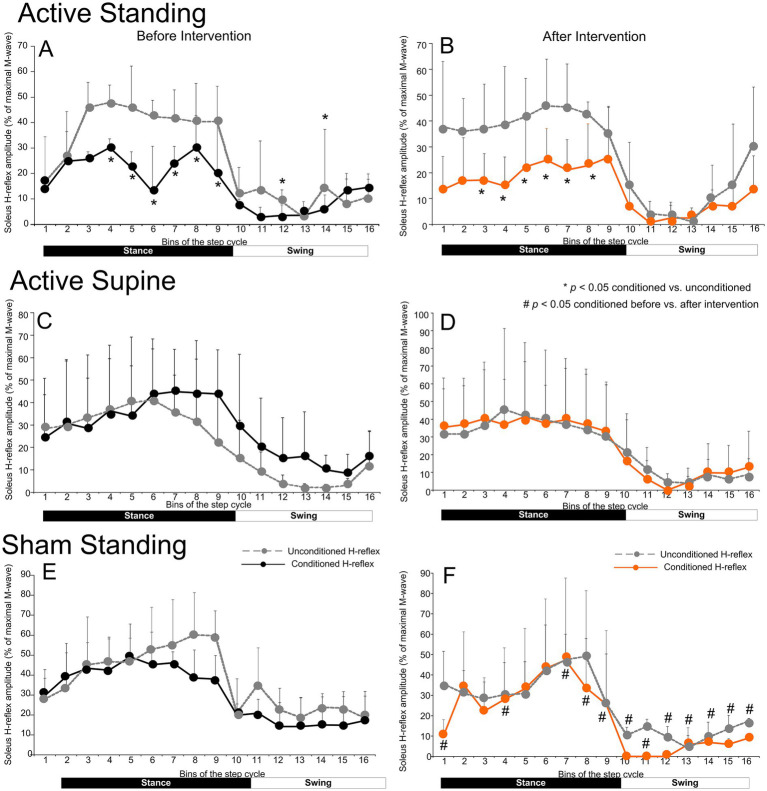
Presynaptic inhibition of soleus Ia afferents before and after each intervention. The mean amplitudes of the conditioned and unconditioned soleus H-reflexes recorded before and after each intervention during stepping are shown as a percentage of the associated maximal M-wave elicited after the unconditioned or test H-reflex at each bin of the step cycle from all subjects. The step cycle was divided into 16 equal bins. Bin 1 corresponds to heel contact. Bins 9, 10, and 16 correspond approximately to stance-to-swing transition, swing phase initiation, and swing-to-stance transition, respectively. Asterisks and hashtags indicate statistically significant differences between conditioned and unconditioned H-reflexes, and between conditioned H-reflexes before and after each intervention, respectively. Error bars in all graphs denote the SD.

The conditioned soleus H-reflexes with CPN stimulation at a 60 ms C-T interval, which reflect the amount of presynaptic inhibition across the step cycle for each study group, are shown in Figure 6.

In the active standing group (*n* = 4), the conditioned H-reflexes were significantly different across bins (*F*_15, 86_ = 5.17, *p* < 0.001; $\eta_{p=}^{2}$ 0.47) but not between before and after the intervention (*F*_1, 86_ = 1.34, *p* = 0.25; $\eta_{p=}^{2}$ 0.015). Furthermore, the conditioned H-reflexes were significantly different from the unconditioned reflexes (*F*_1, 150_ = 42.32, *p* < 0.001; $\eta_{p=}^{2}$ 0.22) at bins 4 to 10 and at bins 12, 14, and 16 (Holm-Sidak; *p* < 0.05), supporting a generalized reflex depression elicited by the conditioning stimulus that remained stable after the intervention.

A similar result was also found for the active supine group (*n* = 4), where the conditioned H-reflexes were significantly different across bins (*F*_15, 112_ = 4.13, *p* < 0.001; $\eta_{p=}^{2}$ 0.35) but not between before and after the intervention (*F*_1, 112_ = 0.84, *p* = 0.36; $\eta_{p=}^{2}$ 0.007). The conditioned H-reflexes were significantly different from the unconditioned reflexes (*F*_1, 175_ = 4.45, *p* = 0.036; $\eta_{p=}^{2}$ 0.02) before training from mid-stance to late swing, but not after training.

In the sham standing group (*n* = 3), the conditioned H-reflexes were significantly different across bins (*F*_15, 64_ = 5.33, *p* < 0.001; $\eta_{p=}^{2}$ 0.55) and after treatment (*F*_1, 64_ = 13.19, *p* < 0.001; $\eta_{p=}^{2}$ 0.19) at bins 1, 4, and 6–16 (Holm-Sidak; *p* < 0.05). Further, the conditioned H-reflexes were significantly different from the unconditioned reflexes (*F*_1, 126_ = 8.2, *p* = 0.005; $\eta_{p=}^{2}$ 0.06) at bins 3, 5, and 7–16 (Holm-Sidak; *p* < 0.05) before and after the intervention (*t* = 3.9, *p* < 0.001).

## Discussion

In this pilot sham-controlled randomized clinical trial, we demonstrated, to our knowledge, for the first time, significant changes in soleus H-reflex phase-dependent amplitude modulation that coincided with changes in reflex excitability gain and threshold in people with chronic SCI who received active transspinal stimulation preceding locomotor training. Furthermore, for those who received active stimulation in the standing position, but not for those who received stimulation in the supine position, reciprocal inhibition during stepping improved after treatment, reflected as reduced ankle co-contraction. Premotoneuronal control of soleus motoneurons was also altered, indicating that our treatment protocol induced meaningful reorganization of spinal neuronal networks.

During walking, functional engagement of spinal neuronal circuits is partly reflected in the close regulation of the soleus H-reflex amplitude at each phase of the step cycle (Knikou et al., 2009a; Nielsen, 2016), a pattern preserved during stepping with a Lokomat (Knikou et al., 2011). Collectively, the soleus H-reflex amplitude increases progressively from early to mid-stance while reflex excitability remains stable during mid stance and late stance, is significantly depressed at the swing-to-stance transition, and remains depressed during the swing phase (Capaday and Stein, 1986, 1987; Simonsen et al., 1995). After SCI, regardless of the severity, a common pathology is a lack of soleus H-reflex depression during the swing phase, and a disruption of sustained reflex excitability during the stance phase (Knikou et al., 2009a; Knikou, 2013).

In the active standing group, soleus H-reflex depression during the mid-swing re-emerged after the intervention (Figure 1A). In contrast, in the active supine group, potentiation during reflex excitability during early- and mid-stance phases was evident after treatment (Figure 2A), with no significant changes observed in the sham standing group (Figure 3A). These results suggest strengthened spinal inhibitory networks during the swing phase, promoting ankle dorsiflexion in the active standing group, and of increased soleus motoneuron depolarization during the stance phase, promoting step progression in the active supine group. Increased reciprocal and presynaptic inhibition of soleus Ia afferent terminals projecting onto soleus alpha motoneurons accounts for the decreased soleus H-reflex excitability during the swing phase (Pratt and Jordan, 1987; Capaday and Stein, 1987; Lavoie et al., 1997; Gosgnach et al., 2000). The enhanced H-reflex amplitude during the stance phase may be mediated by the reversal of weak-to-strong excitatory postsynaptic potentials (EPSPs) with added excitation from Ib interneurons (Capaday and Stein, 1986; Angel et al., 1996) and by stabilization of synaptic inputs to motoneurons (Petruska et al., 2007).

Moreover, the changes in soleus H-reflex phase-dependent modulation in the active standing group occurred with a more linear relationship between the soleus H-reflex and SOL background EMG activity, while the slope increased and the intercept decreased (Figures 1F,G). The increased reflex gain, or the slope of the line relating to H-reflex amplitude and EMG activity level across the whole step cycle, suggests increased reflex sensitivity and, thereby, a decreased reflex threshold. The decreased threshold implies that group Ia muscle spindle afferents became more excitable, altering the recruitment of alpha motoneurons. A tendency for a decreased excitability threshold was also present in the active supine group (Figure 2G), although it was not significant.

We have recently provided evidence from the same study groups regarding changes in reciprocal and presynaptic inhibition at rest (Sayed Ahmad et al., 2025). The reciprocal Ia inhibition exerted from TA onto soleus motoneurons at rest did not change in the active standing group but was strengthened in the sham standing and active supine groups (Sayed Ahmad et al., 2025). In contrast, when we assessed this neuronal mechanism during BWS stepping, we found that only in the active standing group were the conditioned H-reflexes more suppressed during the swing phase compared to the unconditioned H-reflexes, while the reciprocal inhibition was reduced at mid-stance and the swing-to-stance transition, supporting the much-needed increased reflex excitability at these phases. Moreover, the reduced ankle co-contraction in this group (Figure 5A) further supports changes in the strength of reciprocal inhibition. Our findings are consistent with the decreased reciprocal inhibition in early stance or late stance following locomotor training alone in people with SCI (Knikou et al., 2015). Reciprocal Ia inhibition in healthy humans increases mostly during the swing phase of walking (Lavoie et al., 1997; Petersen et al., 1999) but because soleus H-reflex depression during the swing phase is present 50 ms before the onset of TA EMG activity (Crone et al., 1987), is maintained during tonic contraction of ankle extensors in humans (Yang and Whelan, 1993). Ia inhibitory interneurons are active in a phasic pattern when limbs are immobilized in the mesencephalic cat (Feldman and Orlovsky, 1975), it is most likely that this behavior cannot be merely attributed to the alternated EMG activation of ankle flexors and extensors and their background excitability but rather to a central spinal mechanism.

Reciprocal Ia inhibition is prone to presynaptic inhibition (Enríquez-Denton et al., 2000), a powerful regulatory mechanism for sensory afferent feedback-driven excitation during walking. The presynaptic inhibition assessed at rest in the same study groups was potentiated regardless of whether active or sham transspinal stimulation primed locomotor training at rest or during standing (Sayed Ahmad et al., 2025), suggesting that changes in presynaptic inhibition may result from locomotor training rather than from transspinal stimulation. When we assessed presynaptic inhibition during assisted stepping, we found that, in the active standing group, the results supported a generalized reflex depression elicited by the conditioning stimulus that remained unchanged after treatment. While no significant effects were observed in the active supine group, significant differences between the conditioned H-reflexes after treatment and the unconditioned H-reflexes support generalized reflex depression in this group. Thus, we conclude that weight-bearing standing and locomotor training affected presynaptic inhibition more than transspinal stimulation itself. Locomotor training alone reduces presynaptic facilitation or replaces it with presynaptic facilitation at rest, whereas, during stepping, it promotes phase-dependent amplitude modulation in individuals with SCI (Knikou and Mummidisetty, 2014). As the efficacy of transmission in muscle afferents and thereby the strength of their depolarization is under continuous modulation during walking (Gossard et al., 1991), the conditioning TA afferent volleys were most likely not constant but rather were modulated along with the soleus Ia afferents.

Both reciprocal and presynaptic inhibition are prone to modulation by supraspinal centers (Iles and Pisini, 1992). Furthermore, transspinal stimulation uses common motor pathways to those of corticospinal volleys (Knikou, 2014) and alters corticospinal excitability in people with and without SCI (Murray et al., 2019; Murray and Knikou, 2017). Therefore, we can infer that the neurophysiological changes may not be attributable exclusively to the neuronal pathways we studied in this study rather than to integrated changes occurring simultaneously along the neural axis due to locomotor training and transspinal stimulation.

A question that arises is whether, although transspinal stimulation was delivered before locomotor training in resting subjects, the observed neurophysiological changes were specific to step-cycle phases. Transspinal stimulation may have unmasked the inherent spinal gating by acting as a neuromodulatory “primer,” shifting resting membrane potentials and lowering the activation thresholds of spinal interneuronal networks, including Ia inhibitory interneurons (Bolzoni and Jankowska, 2015; Jankowska, 2017; Jankowska and Hammar, 2021). However, over years of walking prior to SCI, these primed pathways became fundamentally state-dependent. They require dynamic, phase-specific peripheral sensory feedback generated during actual walking (e.g., load receptor activation during stance, hip flexor stretch during swing) to function. Therefore, the stimulation likely did not create a phase-specific pattern; rather, it enhanced the excitability of damaged spinal circuits, allowing the spinal cord’s intrinsic, phase-dependent gating mechanisms to operate more effectively during assisted stepping.

The neurophysiological changes we report here with our current intervention paradigm have not been reported in the literature and thus comparison with other studies is not feasible. However, we and others have shown that locomotor training or transspinal stimulation administered separately in people with chronic SCI or animals produces notable changes in reflex excitability, presynaptic and postsynaptic inhibition (Knikou, 2013; Knikou and Mummidisetty, 2014; Knikou et al., 2015; Knikou and Murray, 2019; Minassian et al., 2024; Malloy et al., 2025), further supporting our thesis that transspinal stimulation can augment the benefits of locomotor training.

## Limitations

The main limitation of this randomized sham-controlled clinical trial was the small number of participants who completed the study in each group. This resulted in an imbalance of AIS grades and SCI chronicity across groups (Table 1). For example, two AIS B participants were enrolled in the sham standing group, whereas four AIS D participants were enrolled in the active standing group, resulting in higher baseline function and greater spinal neuronal network function. Moreover, the chronicity of the injury varied among groups with averages of 4.25 ± 3.86, 10.2 ± 4.43, and 9.7 ± 9.5 years in the active standing, sham standing, and active supine groups, respectively. Furthermore, because we did not formally assess masking effectiveness at study completion, we cannot rule out the potential role of expectancy effects. Based on these factors, we suggest that future trials be stratified by AIS lesion grade and injury chronicity and that our results need be interpreted with caution.

## Conclusion

This pilot randomized clinical trial assessed the effects of a 40-session locomotor training primed with active or sham lumbar transspinal stimulation in people with chronic incomplete SCI. Participants were randomized to receive active stimulation in the supine or standing position and to sham stimulation during BWS standing before locomotor training, within the same session. Although limited by the small number of participants, we observed evidence of reorganized spinal neuronal networks after the intervention. These findings indicate the potential for multimodal transspinal stimulation and locomotor training to improve the function of maladaptive spinal networks after chronic SCI.

## Data Availability

The original contributions presented in the study are included in the article/supplementary material, further inquiries can be directed to the corresponding author.
